# The Mycotoxins T-2 and Deoxynivalenol Facilitate the Translocation of *Streptococcus suis* across Porcine Ileal Organoid Monolayers

**DOI:** 10.3390/toxins16090382

**Published:** 2024-09-01

**Authors:** Xiaonan Guan, Arabela R. Martinez, Marcela Fernandez, Francesc Molist, Jerry M. Wells, Regiane R. Santos

**Affiliations:** 1 Schothorst Feed Research, 8212 NA Lelystad, The Netherlandsrsantos@schothorst.nl (R.R.S.); 2 Host-Microbe Interactomics Group, Animal Sciences Department, Wageningen University, 6700 AH Wageningen, The Netherlands; arabela.ritchiemartinez@wur.nl (A.R.M.); marcela.fernandez@wur.nl (M.F.); jerry.wells@wur.nl (J.M.W.)

**Keywords:** intestinal organoids, bacterial translocation, intestinal integrity, *Fusarium* mycotoxins

## Abstract

Mycotoxins have the potential to increase the risk of airway or intestinal infection due to their effects on epithelial integrity and function. The bacterium *Streptococcus suis (S. suis)* is often carried in pigs and can cause outbreaks of invasive disease, leading to sepsis and meningitis in postweaning piglets. In this study, we tested the effect of two *Fusarium* mycotoxins (deoxynivalenol (DON) and T-2) on the integrity of the intestinal epithelium and their interaction with *S. suis*. Porcine ileal organoids were exposed to DON and T-2 individually or in combination and co-cultured with or without *S. suis.* Both DON and T-2 were toxic for ileal organoid monolayers at a concentration of 1 µM but not *S. suis*, even at a higher concentration of 4 µM. To mimic sub-clinical exposures on farms, DON was tested at a concentration of 0.1 µM and T-2 at a concentration of 0.01 µM. The mycotoxins alone did not affect cell permeability, but in combination with *S. suis* there was an increase in epithelial permeability. Furthermore, DON and T-2 together decreased the transepithelial electrical resistance and increased bacterial translocation.

## 1. Introduction

*Streptococcus suis* disease of pigs was first reported in 1954 and is now one of the most important bacterial pathogens in the swine industry worldwide with typical disease symptoms being arthritis, meningitis, and sepsis [1,2]. *S. suis* is also considered an emerging zoonotic pathogen causing similar disease symptoms in humans [3]. Population genomics reveal the presence of diverse commensal lineages of *S. suis* and 10 distinct pathogenic lineages that emerged via a combination of vertical inheritance from a common ancestor and horizontal acquisition of genes both from outside of *S. suis* and from other pathogenic lineages [4]. Lineage 1 containing mostly Serotype 2 strains is responsible for most disease in pigs and humans [4].

Recent research by Fredriksen et al. [5] showed that the tonsil-associated microbiota and colonization by *S. suis* is established soon after birth. This may be attributed to the transmission of bacteria from the vaginal tract during farrowing or via contact with sow saliva. Metagenomic sequencing of DNA extracted from tonsil swabs of healthy asymptomatic piglets revealed that both commensal and pathogenic lineages *S. suis* are present in the tonsil-associated microbiomes [5].

The palatine tonsils and upper respiratory tract are considered the primary sites for *S. suis* to enter the body because the stomach acid constitutes a very efficient barrier against oro-gastrointestinal *S. suis* infections [6]. However, Warneboldt et al. [7] highlighted the fact that this barrier might not function as effectively during times of stress, such as the weaning transition [8,9]. In support of this idea, Su et al. [10] found a significant increase in *S. suis* in the gastrointestinal tract (GIT) of piglets shortly after weaning. Inoculation of *S. suis* into the proximal jejunum by means of a silicon canula was shown to lead to systemic disease in a proportion of challenged piglets showing that intestinal route infection is feasible [11].

*Fusarium mycotoxins* in feed compromise the intestinal barrier, especially in young animals [12]. A recent global survey revealed that 88% of feed for fattening pigs was contaminated with deoxynivalenol (DON), and other mycotoxins [13]. The authors also noted that type B trichothecene DON and type A trichothecene T-2 toxin were present in 45% of the contaminated feed [13]. DON and T-2 toxins compromise the intestinal barrier at relatively high concentrations and inhibit protein synthesis. Additionally, mycotoxins can modulate the immune system, making the pig less likely to respond to vaccinations and increasing the risk for other diseases and infections [14]. Both DON and T-2 toxin disrupt the intestinal barrier [15], aggravate intestinal inflammation caused by infections [16,17], and increase the paracellular passage of drugs and microorganisms [14,18,19].

DON has a high oral availability (48–65%) and is rapidly absorbed by pigs [20]. There is a linear correlation between the dietary intake of DON and serum levels in pigs, where an intake of 0.9 mg DON/kg diet results in circa 7 ng/mL DON in serum [21]. Based on the occurrence data reported from 2007 to 2014 in Europe, the dietary exposure of pigs to DON was calculated as 12.1–54.4 µg/kg b.w. per day [22]. T-2 is difficult to measure in blood due to its rapid metabolism [23], but a previous study showed that 20 min after intravenous administration of T-2 toxin at dosages ranging from 300 to 1200 µg/kg, plasma levels ranged from 60 to 300 µg/kg. After intravenous administration of T-2 toxin (0.15 mg/kg b.w.) to pigs, 15–24% was detected in the gastrointestinal tract [24]. Usually, pig diets are contaminated with T-2 toxin at levels close to 100 µg/kg [25,26]. Therefore, the circulating T-2 toxin levels in pigs are expected to be much lower than those observed by Beasley et al. [23] and Corley et al. [24]. It was estimated that dietary exposure to T-2 and HT-2 toxins in piglets, fattening pigs, and lactating sows ranges from 0.21 to 1.39 µg/kg b.w. per day [27], which is at least 10-times lower than that reported for DON [22]. DON is absorbed either by passive diffusion or via active transport [28], whereas the absorption of T-2 is transporter independent [29].

The hypothesis behind this study is that the co-exposure of intestinal cells to *S. suis* and *Fusarium mycotoxins* increases the translocation of *S. suis* even when the toxins are present at levels that do not cause cell death. The *S. suis* strain P1/7 (SS2) used in this study is internalized by epithelial cells at a low level and was added to primary organoid cells in combination with *Fusarium* mycotoxins to see whether it affected bacterial translocation. In this study, ileal porcine organoids were used as a model because they more closely resemble the in vivo intestinal cell type complexity and functions compared to immortalized cell lines [30]. Furthermore, methods have been developed for generating polarized epithelial monolayers from porcine intestinal organoids [31]. Here, we describe the effects of exposing 2D monolayers to DON, T-2, and *S. suis* on the epithelial viability and permeability and the translocation of *S. suis*.

## 2. Results

### 2.1. Dose–Response of Organoids to DON and T-2 Toxin

**Figure 1:**
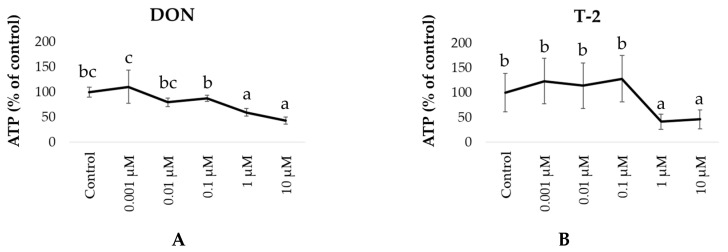
Mean (± SD) viability of porcine 2D monolayers of ileal organoids exposed to DON or T-2 toxin. Organoids were exposed to 0.1–10 µM DON (**A**) or T-2 (**B**) for 24 h. At the end of incubation, the ATP was quantified using the CellTiter-Glo^®^ 2.0 assay. ATP measurements for each treatment are expressed as the percentage of the control value (untreated). a–c Different letters indicate a significant difference among treatments within each tested mycotoxin (*p* ≤ 0.05). The experiment was carried out in triplicate with four independent repetitions.

Initially, 2D monolayers of ileal organoids were exposed to DON or T-2 toxin at concentrations ranging from 0.001 to 10 µM for 24 h to determine the effects on cell viability using the CellTiter-Glo^®^ 2.0 assay. Cellular ATP was significantly decreased in the ileum organoid monolayers exposed to DON or T-2 toxin at a concentration of 1 µM or higher, compared to the untreated control (Figure 1). Further experiments were performed using 0.1 µM DON and 0.01 µM T-2 to reflect the concentrations found in vivo without reducing cell viability in vitro (Figure 1).

### 2.2. DON and T-2 Do Not Inhibit Growth of S. suis

The mycotoxins DON and T-2 toxin did not inhibit the growth of *S. suis* at the highest tested concentration, i.e., 4 µM. As expected, low concentrations of amoxicillin with an MIC of 0.1 µg/mL inhibited *S. suis* (Table 1).

### 2.3. Effect of Conditioned Organoid Medium on Viability of S. suis

It was apparent that organoid-conditioned media derived from ileum organoid monolayers impacted the growth and viability of *S. suis*. There were >30 × 10^8^ CFU/mL with an OD600 of 0.513 in Todd–Hewitt broth (THB) + 0.2% yeast extract at the start of the culture. Approximately 1 × 10^6^ CFU of *S. suis* was added to conditioned and unconditioned Intesticult^TM^ medium and incubated at 37 °C for 18 h. *S. suis* grown in non-conditioned IntestiCult^TM^ had 21.8 × 10^8^ CFU/mL after 18 h of culture. The final CFU/mL of *S. suis* was only 42 × 10^7^ CFU/mL (5-fold lower) when it was grown in organoid-conditioned media (*p* < 0.005).

### 2.4. Permeability of the Cell Monolayer

**Figure 2:**
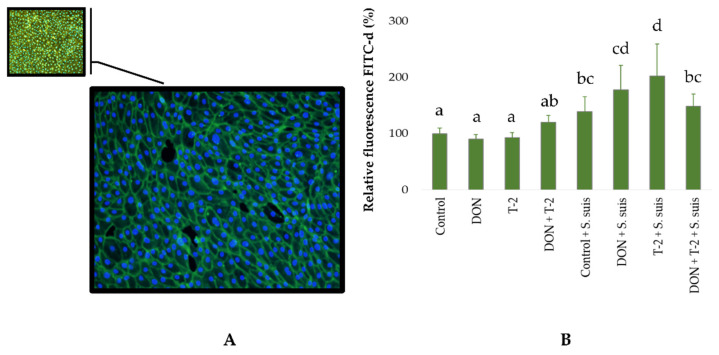
(**A**): Representative ileal organoid monolayers were stained with Hoechst (nuclei in blue) and phalloidin (actin stain in green) (**B**): Mean (± SD) permeability to 4 kDa FITC-dextran was quantified by measurement of fluorescence in the basal compartment after 18 h and indicated as a percentage of the mean for the untreated control. The different letters a–d significant differences among treatments (*p* ≤ 0.05). The experiment was carried out in triplicate with four independent repetitions.

Figure 2 depicts representative images of ileal organoid monolayers at the start of the culture, with confluent monolayer in all treatments (Figure 2A). The permeability of the cell monolayer to 4 kDa FITC-dextran was measured as described in the Materials and Methods and calculated as shown relative to the permeability of the untreated control monolayers (Figure 2B). Exposure to mycotoxins did not significantly increase the permeability of the ileum organoid monolayers. However, the exposure to 1 × 10^6^ CFU/mL *S. suis* in the control treatment led to an increase in permeability, which was further increased in combination with 0.01 µM T-2 (*p* < 0.01) (Figure 2B).

### 2.5. Effects of Mycotoxins and S. suis on Transepithelial Electrical Resistance

**Figure 3:**
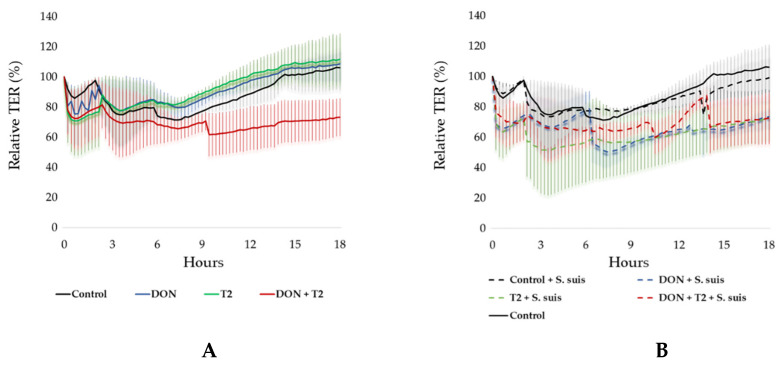
Mean (± SD) transepithelial electric resistance (TER) of a 2D organoid culture continuously measured for 18 h post-seeding. (**A**) Treatments without *S. suis* (Control, DON, T2, and DON + T2); (**B**) Control group without *S. suis* (Control) and mycotoxins combined with *S. suis* exposure (Control + *S. suis*, DON + *S. suis*, T2 + *S. suis*, and DON + T2 + *S. suis*). The experiment was carried out in triplicate with four independent repetitions.

At the start of the experiment, the TER values of organoid monolayers varied slightly but gave a minimum TER value of 400 Ω/cm^2^, indicating confluence. Changes in TER due to the different treatments were expressed as a percentage of the starting value at time zero (100%). At the start of the experiment, the TER values of the controls initially dropped to around 80% of the starting value and then gradually increased to 100% of the initial value over 18 h. A similar profile was observed when only 0.1 µM DON or 0.01 µM T-2 toxin was added to the apical culture medium (Figure 3A). However, the combination of 0.1 µM DON with 0.01 µM T-2 toxin resulted in lower TER values over 18 h (Figure 3A). The addition of *S. suis* alone did not significantly decrease the TER over time, but in combination with 0.1 µM DON and 0.01 µM T-2 toxin, or the combination of mycotoxins, the TER value remained lower than 60% of the starting value between 6 and 18 h (Figure 3B) (*p *≤ 0.05).

### 2.6. Translocation of S. suis from the Apical to the Basal Compartment

**Figure 4:**
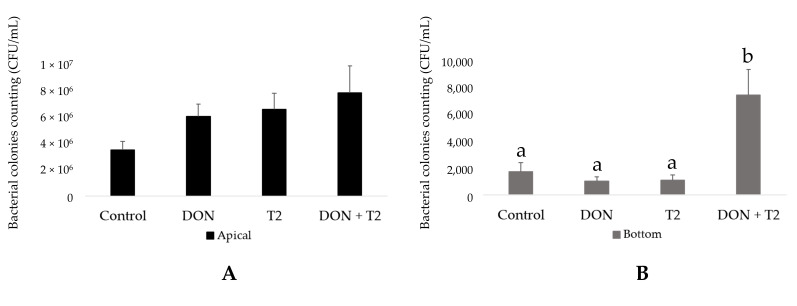
Mean (± SD) *S. suis* colony forming units/mL (CFU/mL) in the apical (**A**) and basal (**B**) compartments of the culture wells. a,b Different letters indicate significant differences among treatments within the basal part (*p* ≤ 0.05). DON: 0.1 µM; T-2: 0.01 µM; DON + T-2: 0.1 µM + 0.01 µM, respectively. The experiment was carried out in triplicate with four independent repetitions.

Conditioned medium from ileum organoid monolayers was found to inhibit the growth of *S. suis* during the 18 h culture, although surviving bacteria could be recovered by plating. The addition of mycotoxins did not affect the growth of *S. suis* in the apical part of the wells compared to *S. suis* alone (control). The addition of 0.1 µM DON and 0.01 µM T-2 together significantly increased the CFU of *S. suis* in the basal compartments (Figure 4A) compared to the controls and individual mycotoxin treatments (*p* < 0.0001) (Figure 4B). As expected, no colonies were observed in the culture wells not inoculated with *S. suis*.

## 3. Discussion

Initially, we tested the effects of the mycotoxins DON and T-2 toxin on the viability of ileal organoid monolayers at a range of concentrations, from 0.001 to 10 µM. A decrease in mitochondrial activity was observed after exposure to DON or T-2 toxin, at a concentration of 1 µM. This is likely due to the known toxic effects of DON and T2 on mitochondria, leading to oxidative stress, impaired mitochondrial function, and the disruption of ATP production, ultimately leading to cell death [32]. This concentration is close to the IC_50_ values of DON for IPEC-1 cells (1.3 µM) but three times lower than that for IPEC-J2 cells (3 µM) [33]. The cytotoxic concentrations of T-2 toxin are 0.005 µM for IPEC-J2 [34], 0.093 µM for IPEC-1 cells [35], and 0.015 µM for intestinal explants [35]. Both IPEC-1 and IPEC-J2 are intestinal epithelial cell lines derived from the jejunum of unsuckled newborn piglets younger than 12 h old [36]. The differences in IC_50_ for cell lines and stem cell-derived epithelial cells may be due to metabolism and anomalies commonly associated with immortalized cell lines [37,38,39]. In contrast, the small intestinal organoid monolayers contain all cell lineages present in the intestinal epithelium and have a similar transcriptome landscape to the tissue in vivo [31].

Based on the concentration of mycotoxins reducing cell mitochondrial activity (Figure 1), we chose to use 0.1 µM DON and/or 0.01 µM T-2 toxin for further studies. The tested concentrations were also selected based on other considerations, including the reported DON serum levels in pigs after oral exposure to DON [21], the serum levels of T-2 toxin after intravenous administration [23,24], the expected DON and T-2 toxin levels in the final diet of pigs [21,26], the daily exposure to DON and T2 [22,27,40], and other studies using IPEC-1 and IPEC-J2 cells [18,33,35]. For T-2 toxin, supposing a dietary intake of 100 µg/kg and a recovery of 5% in serum, the anticipated circulating concentration would be 0.5 ng/mL, corresponding to approximately 0.01 µM of T-2 toxin. Furthermore, the selected concentrations did not affect *S. suis* growth. The antibacterial activity of DON and T-2 toxin was tested against several bacterial species from the genera *Streptococcus*, *Escherichia*, *Staphylococcus*, *Yersinia*, *Salmonella*, *Erysipelothrix*, and *Lactobacillus* [41]. These authors observed that the growth of *Streptococcus agalactia* was inhibited after exposure to very high levels (160 µg/mL) of DON (i.e., 0.54 mM) or T-2 (0.36 mM).

In this study, ileal organoid monolayers were simultaneously exposed to *S. suis* and both mycotoxins for 18 h. The growth of *S. suis* in the apical chamber was very slow compared to the bacteriological medium, likely due to the production of host antimicrobial peptides. Therefore, 18 h of co-culture with *S. suis* did not result in bacterial overgrowth. After 18 h of co-culturing *S. suis* in the apical chamber of confluent ileum monolayers in transwell devices, the permeability to fluorescent dextrans increased. This is likely due to the activity of secreted suilysin, a cholesterol-dependent cytolysin that can have dose-dependent detrimental effects on cells [42,43]. A similar study using tracheal epithelium showed the translocation of S. suis into the basal chamber [43]. The action of suilysin may explain the bacterial translocation observed in all treatments, including the control medium containing *S. suis* without mycotoxins. Importantly, *S. suis* translocation was significantly increased when 0.1 µM DON and 0.01 µM T-2 toxin were added to the culture medium. Although the combination of both DON and T-2 toxins did not affect the permeability of the cell monolayer to FITC-dextran, a significant decrease in TER was observed when organoid monolayers independent of the presence of *S. suis* in the culture. On the other hand, assessments of permeability using FITC-dextran showed that *S. suis* increases permeability, but *S. suis* alone did not significantly affect the TER. The decrease in TER values reflects an increase in the paracellular ion permeability of the monolayer, but this does not necessarily equate to the increased permeability of small molecules, indicating that it is possible to modulate one of these parameters without altering the other [44]. Although FITC-d and TER can assess barrier function, Zucco et al. [45] demonstrated that these approaches can lead to different outcomes depending on the involved transport mechanisms. For instance, FITC-d is used to assess the non-electrolyte passage of macromolecules based on increased paracellular flow and the pore size of tight junctions, whereas a decreased TER indicates ionic conductance [45], which may have played a major role in the bacterial translocation observed in the present study. The *S. suis* isolate of SS2 clonal complex 1 (CC1) has been shown to translocate across polarized IPEC-J2 cells within 6 h via the paracellular route [46]. The presence of 0.1 µM DON and 0.01 µM T-2 toxin in the medium may exacerbate the translocation of *S. suis.* There are several studies demonstrating that DON promotes the translocation of other bacteria, including *Salmonella typhimurium* (exposure of IPEC-J2 cells to 148 mM DON for 24 h followed by 1 h of incubation with *S. typhiurium*) [47] and after feeding broiler chickens with diets contaminated with DON and exposed to *Escherichia coli* [48] and *Campylobacter jejuni* [49]. Similarly, the exposure of IPEC-J2 cells to 460 µM T-2 toxin for 24 h led to the translocation of Salmonella typhimurium when added for one hour [19].

*In vivo*, bacterial translocation via the paracellular route in the intestine will depend on the pig’s health status and exposure to stress conditions [50]. *S. suis* has been identified in the content of the small intestine but typically in low numbers [7]. This study suggests that chronic exposure to low amounts of the mycotoxins DON and T-2 can facilitate the translocation of *S. suis* and potentially inflammatory components of other bacteria across the ileal epithelium.

## 4. Conclusions

We conclude that concentrations of 0.1 µM DON and 0.01 µM T-2 toxin together can decrease the TER of the ileal organoid monolayer. The tested concentrations of mycotoxin are similar to those measured in the porcine plasma of pigs with a moderate dietary exposure to these mycotoxins. In combination with the mycotoxins, the translocation of *S. suis* across the epithelium was increased. Our findings suggest that chronic exposure to mycotoxins may result in infections or heightened inflammation due to decreased barrier function which increases the risk for welfare issues and mortality. The model described has applications for testing solutions to improve intestinal barrier function.

## 5. Materials and Methods

### 5.1. Chemicals and Culture Media

Chemicals were purchased from Sigma (St. Louis, MO, USA) unless stated otherwise. DON (purity > 97%) and T-2 (purity > 97%) were dissolved in DMSO at a concentration of 1 mM. Aliquots of this stock solution were stored at −20 °C. On the day of the experiments, the stock solution was diluted to the final concentrations in the culture medium and sonicated for 15 min. The final concentration of DMSO in all culture media was 0.01%. Culture media were equilibrated at 5% CO_2_ and 37 °C for at least two hours before use.

### 5.2. Bacterial Strain and Culture Condition

The *S. suis* serotype 2 (SS2) P1/7, the pathogenic European reference strain [AM946016] [51], was obtained from Wageningen Bioveterinary Research. The bacterium was cultured overnight at 37 °C under aerobic conditions in Todd–Hewitt broth (Oxoid Ltd., Basingstoke, UK) with 0.2% yeast extract. The bacterium was then recovered by centrifugation and washed twice with phosphate-buffered saline (PBS). The final concentration of bacterial culture was diluted to reach an OD600 of 0.005, which corresponded to 1 × 10^6^ colony-forming units (CFU)/mL. The exact number of CFU was confirmed by plating serial dilutions (10^−3^, 10^−4^, 10^−5^) on agar plates produced from THB supplemented with 0.2% yeast extract and 1.5% agar (BD Difco^TM^, Franklin Lakes, NJ, USA) and cultured for 24 h at 37 °C in the presence of 5% CO_2._

### 5.3. Minimum Inhibitory Concentration of DON and T-2 on S. suis

The minimum inhibitory concentrations (MIC) of DON and T-2 were determined using the *S. suis* serotype 2 P1/7, following the Clinical and Laboratory Standards Institute (CLSI) Standard Broth Micro-dilution Method with minor modifications. Briefly, serial two-fold dilutions of 4 µM DON or 0.32 µM T-2 in PBS+ were made down to the final concentrations of 0.0312 and 0.003 µM DON and T-2, respectively, in a U-bottom 96-well plate (100 µL per well). To each well, 100 µL of 10^6^ CFU/mL of the bacterial suspension was added, resulting in a final volume of 200 µL and a final concentration of 10^5^ CFU/mL. Wells with sterile PBS+ alone served as blanks. Wells with serial two-fold dilutions of amoxicillin in a range of 0.02 ng/mL to 200 µg/mL served as positive controls. Plates were incubated at 37 °C for 24 h. Thereafter, OD values were measured at a 655 nm wavelength after transferring 100 µL of the incubated suspension to a new sterile flat-bottom 96-well plate. The MIC was defined as the lowest mycotoxin (DON or T-2) concentration, resulting in an OD value similar to the blank (PBS+).

### 5.4. Ileum Organoid Culture

For ileal organoids, spheroids at 5 days after passaging were recovered from Matrigel and dissociated into single cells as previously described [31]. Briefly, 3D organoids were transferred to 15 mL Falcon™ tubes and pelleted for 5 min at 300× *g*. Subsequently, the pellets were resuspended in TryplE™ (Gibco, Thermo Fischer Scientific, Inc., Waltham, MA, USA) and incubated for 5–10 min at 37 °C. Trypsinization was stopped by adding ice-cold DMEM/F12 containing 10% fetal calf serum (Gibco, Thermo Fischer Scientific) followed by centrifugation for 5 min at 450× *g*. Cells were resuspended in growth media (IntestiCult™ Organoid Growth Medium, STEMCELL™ technologies, Inc., Vancouver, BC, Canada) and seeded on precoated transwell cell culture inserts (Transwell™, Corning, MA, USA) with a pore size of 3.0 um at a density of 2.5 * 10⁵. The basolateral chamber of the 24-well transwell insert plates was filled with 0.6 mL of growth medium, and the apical chamber was filled with 0.5 mL of growth medium (STEMCELL™ technologies). The ileal organoid monolayer cultures were incubated for up to 5 days at 37 °C with 5% CO_2_, exchanging the medium every third day. Transepithelial resistance (TER) in the transwell cultures was monitored daily until the TER reached around 400 Ω*cm^2^.

### 5.5. Bacterial Viability after Exposure to Organoid Conditioned and Non-Conditioned Media

To study the viability of bacteria in conditioned and non-conditioned organoid media, porcine ileum 2D organoids were cultured with 500 µL of IntestiCult™ without antibiotics for 18 h at 37 °C and 5% CO_2_. The supernatant was then harvested and centrifuged at 500× *g* for 10 min. Overnight THB + 0.2% yeast extract cultures of *S. suis* were centrifuged at 7000 rpm for 5 min and resuspended in Dulbecco’s phosphate-buffered saline (D-PBS). To measure the antibacterial activity of conditioned IntestiCult™, *S. suis* was inoculated in a 24-well plate filled in triplicate with 500 µL of conditioned IntestiCult™, non-conditioned IntestiCult™, and THB + 0.2% yeast extract (at a final concentration of 10^5^ CFU/mL) for a total of 18 h. To enumerate the surviving CFU, 100 µL of suspensions from all samples was removed from the wells and diluted five-fold in sterile D-PBS. The dilutions were then plated using THB + 0.2% yeast solid agar plates for 24 h and bacterial survival was determined by CFU/mL.

### 5.6. Cell Viability

CellTiter-Glo^®^ 2.0 (Promega, Madison, WI, USA) was used to measure ATP produced by the cells after lysis. Porcine ileal organoids were cultured in IntestiCult™ Organoid Growth Medium (STEMCELL) in a 24-well opaque plate until confluency. The cells were subsequently cultured in the same medium containing 0–10 µM DON or 0–10 µM T-2 for 24 h in 5% CO_2_ at 37 °C. After culture, CellTiter-Glo 2.0^®^ Reagent (Promega) was equilibrated to room temperature and added to all wells (0.5 mL per well for 24-well plates). To induce cell lysis, an orbital shaker was used for 2–5 min, after which the plate was incubated at room temperature for 10 min. Immediately after the last step, luminescence was recorded in a Spectramax (Molecular devices). A blank without cells was used as an extra control.

### 5.7. Permeability Measurement (Non-Ionic)

To follow the non-ionic epithelial permeability [44,45], just before starting a culture in a Cellzcope apparatus (Nanoanalytics, Münster, Germany), cells from each well were apically incubated with 0.5 mg/mL 0.4 kDa Fluorescein isothiocyanate-dextran (FITC, 4 kDa FITC-Dextran; Thermo Fisher Scientific). After 18 h of culture, samples from basolateral culture medium were transferred to a 96-well plate and measured for FITC-dextran contents using fluorescence on a Spectramax M5 (Molecular Devices, Sunnyvale, CA, USA) with 490 nm excitation and 530 nm emission. Three wells containing only culture medium and FITC-dextran were used as controls.

### 5.8. Transepithelial Electrical Resistance (Ionic)

To follow the ionic epithelial permeability [44,45], transwell inserts were coated in the apical chamber, and single cells were seeded at a density of 2.5 × 10⁵ cells/well in 24-well transparent transwell inserts (0.33 cm^2^, Falcon, BD) and incubated with 800 μL basolateral culture medium and 400 μL apical culture medium with or without 0.1 µM DON, 0.01 µM T-2, *S. suis*, or various combinations thereof. For transepithelial electrical resistance (TER) measurements, three transwell inserts per treatment were placed in a Cellzscope apparatus (Nanoanalytics, Münster, Germany) 2 h after seeding. The TER was measured over 18 h post-seeding, as previously described [52].

### 5.9. Bacterial Viability in the Apical and Bottom Compartments

To quantify the number of culturable (i.e., actively growing) bacterial cells in the apical and at the bottom of the culture wells after exposure or non-exposure to mycotoxins, a plating method was conducted. For this, 100 µL of supernatant was separately taken from each well and subjected to 10-fold serial dilutions in PBS+ (×10^−5^). From the bottom, 100 µL of the medium was separately taken from each well and subjected to 10-fold serial dilutions in TSB+ (×10^−2^). Subsequently, these diluted samples were plated onto TSA and cultured for 24 h at 37 °C. After culture, the number of bacterial colonies was counted.

### 5.10. Statistical Analysis

The cells (2D ileal organoids) were randomly assigned to the treatment conditions. Statistical analysis was conducted with the GenStat 21 statistical software (GenStat for Windows 21st Edition, VSN International, Hemel Hempstead, UK; https://www.vsni.co.uk/downloads/genstat/; accessed on 12 February 2024). The experiments were carried out in triplicate with four independent repetitions. The null hypothesis was that there was no treatment effect on the response parameter. Treatment means were compared according to Fisher’s LSD (for a two-sided test and *p* ≤ 0.05) after a significant treatment effect was confirmed by ANOVA. Data are presented as the mean ± SEM. The *p*-value of the statistical model is given per response parameter. Effects with *p* ≤ 0.05 were statistically significant.

## Figures and Tables

**Table 1:** Minimum inhibitory concentration (MIC) values for the mycotoxins DON and T-2 and the antibiotic amoxicillin against *S. suis*.

	MIC
DON (up to 4 µM)	No
T-2 (up to 4 µM)	No
Amoxicillin (µg/mL)	0.1

No: no MIC obtained. DON and T-2 were tested at concentrations ranging from 0.03 to 4 µM.

## Data Availability

Data are contained within the article.

## Abbreviations

ANOVA	Analysis of variance
ATP	Adenosine triphosphate
CC1	Clonal complex 1
CFU	Colony forming unit
CLSI	Clinical and Laboratory Standards Institute
DMEM	Dulbecco’s Modified Eagle Medium
DMSO	Dimethylsulfoxide
DON	Deoxynivalenol
EU	European Union
FITC-d	Fluorescein isothiocyanate-dextran
HT-2	HT-2 toxin
IPEC-1	Intestinal porcine epithelial cells 1
IPEC-J2	Intestinal porcine epithelial cells J2
LSD	Least significant difference
MIC	Minimum inhibitory concentration
PBS	Phosphate buffer saline
SEM	Standard error of the means
SS2	*Streptococcus suis* serotype 2
T-2	T-2 toxin
TER	Transepithelial electrical resistance
THB	Todd-Hewitt broth
